# Probing Molecular Mechanisms of the Hsp90 Chaperone: Biophysical Modeling Identifies Key Regulators of Functional Dynamics

**DOI:** 10.1371/journal.pone.0037605

**Published:** 2012-05-18

**Authors:** Anshuman Dixit, Gennady M. Verkhivker

**Affiliations:** 1 Department of Pharmaceutical Chemistry, School of Pharmacy, The University of Kansas, Lawrence, Kansas, United States of America; 2 School of Computational Sciences and Crean School of Health and Life Sciences, Schmid College of Science and Technology, Chapman University, Orange, California, United States of America; 3 Department of Pharmacology, University of California San Diego, La Jolla, California, United States of America; Fred Hutchinson Cancer Research Center, United States of America

## Abstract

Deciphering functional mechanisms of the Hsp90 chaperone machinery is an important objective in cancer biology aiming to facilitate discovery of targeted anti-cancer therapies. Despite significant advances in understanding structure and function of molecular chaperones, organizing molecular principles that control the relationship between conformational diversity and functional mechanisms of the Hsp90 activity lack a sufficient quantitative characterization. We combined molecular dynamics simulations, principal component analysis, the energy landscape model and structure-functional analysis of Hsp90 regulatory interactions to systematically investigate functional dynamics of the molecular chaperone. This approach has identified a network of conserved regions common to the Hsp90 chaperones that could play a universal role in coordinating functional dynamics, principal collective motions and allosteric signaling of Hsp90. We have found that these functional motifs may be utilized by the molecular chaperone machinery to act collectively as central regulators of Hsp90 dynamics and activity, including the inter-domain communications, control of ATP hydrolysis, and protein client binding. These findings have provided support to a long-standing assertion that allosteric regulation and catalysis may have emerged via common evolutionary routes. The interaction networks regulating functional motions of Hsp90 may be determined by the inherent structural architecture of the molecular chaperone. At the same time, the thermodynamics-based “conformational selection” of functional states is likely to be activated based on the nature of the binding partner. This mechanistic model of Hsp90 dynamics and function is consistent with the notion that allosteric networks orchestrating cooperative protein motions can be formed by evolutionary conserved and sparsely connected residue clusters. Hence, allosteric signaling through a small network of distantly connected residue clusters may be a rather general functional requirement encoded across molecular chaperones. The obtained insights may be useful in guiding discovery of allosteric Hsp90 inhibitors targeting protein interfaces with co-chaperones and protein binding clients.

## Introduction

The molecular chaperone Hsp90 (90 kDa heat-shock protein) is required for managing conformational development, stability and function of proteins in the cellular environment [1]–[6]. Molecular chaperones play a pivotal role at the crossroads of multiple signaling pathways associated with cell proliferation and viability, wherein upregulation of their activity can promote tumor cell adaptation. Hsp90 is an important hub in a variety of protein interaction networks associated with oncogenic pathways and responsible for the conformational maturation of proteins [7]–[10]. The repertoire of Hsp90 client proteins entails a wide range of regulatory co-chaperones and signaling molecules, most notably including protein kinases, transcription factors, and overexpressed signaling proteins involved in the control of cell homeostasis, proliferation, differentiation, and apoptosis [11]–[19]. Hsp90 has emerged as one of the most promising biological targets identified for the treatment of cancer since this molecular chaperone is responsible for folding of the proteins directly associated with all six hallmarks of cancer [20]. Mechanism-based anti-cancer agents can act on specific oncogenic proteins hijacked by the pathological genetic and epigenetic changes leading to the initiation of malignancy and cancer progression. As a result, broader therapeutic prospects are typically offered by targeting signaling networks that oversee multiple aspects of tumor cell maintenance. Inhibition of the Hsp90 protein folding machinery can often result in the disruption of numerous oncogenic pathways, while simultaneously achieving tumor cell specificity [21]–[25]. By disabling multiple signaling circuitries, Hsp90 inhibition provides a novel therapeutic strategy in cancer research, selective for specific cancer mechanisms, yet broadly applicable to disparate tumors with different genetic signatures [26]–[35].

Structural and biochemical studies have established Hsp90 as an ATP-dependent system that operates as a homodimer in a functional cycle associated with the ATP binding and hydrolysis [36]–[42]. Upon ATP-mediated dimerization of the N-termini, the activated Hsp90 can assume a closed “clamped” conformation, engulfing the client protein [38]–[40]. Co-chaperone recruitment can facilitate ATP hydrolysis and stabilize Hsp90 allowing for the maturation and subsequent release of the client protein [41], [42]. Hsp90 inhibition can prevent conformational maturation of Hsp90-dependent oncogenic clients and cause abolishment of their oncogenic activity by disabling the Hsp90 complex that then becomes a substrate for subsequent ubiquination and proteasomal degradation [43]. Structural biology studies have been instrumental in progressing understanding of the conformational dynamics and molecular mechanisms of the Hsp90 chaperone [44]–[46]. The initial structural efforts concentrated on isolated, individual domains of yeast Hsp90 [47]–[50], the endoplasmic reticulum (ER) homologue Grp94 [51], [52] and the *E coli* homologue, HtpG [53]. The first X-ray crystal structures of the full-length Hsp90 came from the co-crystal structure of yeast Hsp90 bound to the AMP-PNP and a co-chaperone p23 (mammals)/Sba1 (yeast homologue) [54]. This structure revealed that each Hsp90 protomer was characterized by a modular architecture with three well-defined domains: an N-terminal domain (NTD) responsible for ATP binding, a Middle Domain (M-domain), which completes the ATPase site necessary for ATP hydrolysis and binds client proteins, and a C-terminal domain (CTD) that is required for dimerization [54]. The crystal structures of Hsp90-cochaperone complex from yeast [54], E. coli HtpG [55], Grp94 homologue-nucleotide complex [56] and an Hsp90-client protein complex [57], have revealed the dramatic conformational changes in the molecular chaperones that could accompany the ATPase cycle. A considerable mobility of the molecular chaperone was seen in diverse structural arrangements of the crystallographic conformations, ranging from a structurally rigid and closed ATP-bound form of yeast Hsp90 [54] to a V-shaped Apo-form and a semi-closed ADP-bound form of the bacterial homologue HtpG [55]; and an intertwined conformation with close contacts between the two NBDs in the Grp94 homologue [56]. Structural and biochemical studies supported a mechanism of conformational coupling to the ATPase cycle that is conserved among different species [58] and involves a “tense”, structurally rigid conformational state of Hsp90 upon ATP binding, whereas subsequent hydrolysis to ADP leads to a more “relaxed” state of Hsp90 and, in the nucleotide-free form, the dimer moves to an “open” state. In accordance with this mechanism, the molecular chaperone Hsp90 undergoes a coordinated transient dimerization of the NTDs and association of the NTD and the M-domain in the ATP-bound state, but not in the ADP-bound or apo states. The Hsp90 progression from its “relaxed” conformation to the “tight” complex where the NTDs become closely associated is central to the ATPase cycle and essential for protein client activation.

Modern biophysical approaches including X-ray crystallography, electron microscopy and small-angle X-ray scattering (SAXS) have provided a more detailed characterization of the Hsp90 conformational states during progression through the ATPase cycle [54]–[64]. Structural studies of the isolated Grp94-NTD [51], [52] and the full-length ER homologue Grp94 in complexes with AMP-PNP and ADP [56] have revealed an intermediate ‘twisted V’-like conformation, held together through extensive contacts at the C-terminal interface, that is catalytically silent and conformationally insensitive to the identity of the bound nucleotide. These studies have indicated that nucleotide binding would not induce global conformational rearrangements in Grp94, but rather ligand-driven conformational changes could be limited to the NTD domain [51], [52], [56]. Structural and biochemical studies using crystallographic [55] and SAXS approaches [59] have shown that apo-HtpG may fluctuate between open and extended states, while preserving the constitutive dimerization of the CTDs [60], [61]. Although apo-HtpG favors an open conformation, a highly compact HtpG dimer is formed in the presence of ADP [55]. Interestingly, even in the presence of AMP-PNP, HtpG can still maintain equilibrium between the open and the closed states. However, the extent to which the nucleotide binding can modulate differences between open and closed dimer conformations is much larger for HtpG as compared to the yeast Hsp90 [55], [60]–[62]. Recent studies based on mutational analysis, cross-linking and electron microscopy (EM) in bacteria, yeast, and humans [63], [64] have revealed that the open and closed conformations may co-exist during a dynamic equilibrium in the apo, ADP, and AMP-PNP states across different species. Furthermore, SAXS studies of pH-dependent conformational changes in apo-HtpG have unveiled two-state equilibrium between the extended solution HtpG structure and a second state which was strikingly similar to the crystal structure of the Grp94 dimer homologue [65]. Mass spectrometry investigations of human Hsp90 in solution have shown that co-chaperone and inhibitor binding to the Hsp90-NTD can induce allosteric conformational changes that are consistent with the thermodynamic model of pre-existing chaperone states [66], [67]. Single-molecule fluorescence resonance energy transfer (FRET) experiments [68], [69] have directly monitored the large conformational changes within yeast Hsp90, demonstrating that conformational fluctuations between the open and closed states may be only weakly coupled to the rate of ATP hydrolysis. Conformational dynamics of HtpG in solution has been also studied by using amide hydrogen exchange mass spectrometry (HX-MS) and fluorescence spectroscopy [70]. Remarkably, it has been discovered that in all states of the ATPase cycle the molecular chaperone can continuously fluctuate between two closed and two open conformations. Biochemical and HX-MS experiments have suggested important functional elements of Hsp90 that may serve as key regulators of the chaperone activity [71], [72]. A conserved NTD hydrophobic motif and a charged linker at the boundary between Hsp90-NTD and the M-domain could form one of these regulatory clusters, wherein single or double residue mutations can inhibit Hsp90 function [71]. Mutagenesis and functional studies of Hsp90 regulation have also identified a single conserved residue in the CTD shared by the chaperone family as a strong regulator of Hsp90 functions, including ATP hydrolysis and chaperone activity [72]. Single-molecule FRET experiments of Hsp90 have allowed to dissect for the first time functionally coordinated kinetics of CTD and NTD upon nucleotide binding, providing a compelling evidence of anti-correlated motions of the C-terminal open and an N-terminal closed state [73]. The occupancy of the C-terminal open conformation was found to be modulated by nucleotides bound to the N-terminal domain, thus providing a strong evidence of long-range communication between the two terminal domains of the molecular chaperone [73]. Collectively, structural and biochemical studies have proposed that molecular chaperones can function using a nucleotide-triggered switching between the apo-open and ATP-closed states, wherein ligand-based modulation of the conformational dynamics can bias the equilibrium towards functionally relevant complexes [45], [46]. According to this mechanism, the ATPase cycle of Hsp90 is not a conformationally deterministic process, but rather a subtle functional regulator of the equilibrium between preexisting conformational states.

Computational approaches have been instrumental in revealing the atomic details of the inter-domain communication pathways which may regulate the conformational equilibrium of the molecular chaperone. We have recently reported the first computational study of the Hsp90 allosteric binding with an atomic level analysis of the conformational motions and the inter-domain communication pathways in the full-length yeast Hsp90 dimer in the apo form and complexes with ATP and ADP [74]–[76]. In these studies, we have found that in the presence of ATP long-range communication from the nucleotide binding site is mainly directed to the specific residues at the CTD dimerization interface, while ADP can activate allosteric signaling between the binding site and residues from the C-terminal region that surround the CTD dimerization interface. These results have provided evidence of a possible cross-talk between N- and C-terminal binding sites of Hsp90 that may induce an allosteric regulation of the molecular chaperone machinery. Hence, computational studies [74]–[76] have confirmed the results of single-molecule-molecule FRET experiments [73] by demonstrating that nucleotide or ligand-based modulation of the conformational dynamics can be communicated over long distances and bias the equilibrium towards functionally relevant complexes. Conformational flexibility can be employed in the molecular chaperone function and adaptation to co-chaperones and protein clients via equilibrium switching towards the preferential conformational state [77]–[79]. Using a combination of SAXS, FRET and NMR experiments, it has been recently shown that protein client binding may serve as a kinetic accelerator of the conformational changes in Hsp90, inducing a partially closed conformation of Hsp90 and enhancing the ATPase activity [78], [79]. Recent studies have also demonstrated that the Hsp90 CTD is important for dimerization of the chaperone and contains a second nucleotide binding site [80]–[82]. Allosteric interactions between Hsp90-CTD and M-domains could mediate Hsp90 recognition of client proteins and promote (or prevent) binding of co-chaperones and immunophillins [83]–[87]. It was conjectured that the inter-domain communication between NTDs and CTDs of the Hsp90 chaperone may trigger a potential transient separation of the CTDs, thus unveiling an otherwise hidden secondary C-terminal binding site [53]–[55]. Biochemical [88]–[93] and computational studies of the Hsp90 C-terminal inhibitors [94]–[96] have shown that allosteric modulators of Hsp90 can induce client protein degradation without induction of the heat shock response. These studies have confirmed that Hsp90 C-terminal inhibitors can bind to the C-terminus and allosterically antagonize the chaperone function by inducing a conformationally favorable separation of the Hsp90 CTDs and thereby interfere with the Hsp90 dimerization [94]–[96].

Mechanistic and thermodynamic models of allosteric communication have become widely accepted and broadly applied to analyze mechanisms of allosteric regulation in proteins and biological networks [97]–[109]. Allosteric communication mechanisms can range from a sequential model, where binding of a molecule at one site causes a sequential propagation of conformational changes across the protein, to an intermediate, “block-based” model of interacting sparse residue clusters, and to a fully cooperative model, where structural changes are tightly coupled [110]. The emerging consensus is that allostery may be inherent to all proteins and involve multiple communication pathways and interaction networks between preexisting conformational states [97]–[109]. All-atom and coarse-grained models [111]–[115] combined with the normal mode analyses [116]–[118] have shown that protein conformational dynamics underlying fundamental biological functions may be largely determined by the native state topology. According to these models, allosteric interactions may be determined by the equilibrium structure and functionally important conformational transitions could be adequately described by the low frequency normal modes [119]–[121]. Furthermore, functional dynamics along low frequency modes is typically evolutionary conserved [122]–[127], and proteins sharing the same fold architecture may exhibit similar dynamical characteristics.

Structure-functional studies of Hsp90 regulation have proposed a number of functional motifs that may contribute to the regulation of Hsp90 activity, including ATP hydrolysis and allosteric interactions. Nevertheless, molecular mechanisms that control the relationship between conformational diversity and biological functions of Hsp90 lack a detailed quantitative characterization. In this work, we integrated molecular dynamics (MD) simulations and modeling of the principal collective motions with the energy landscape models and structure-functional analyses of regulatory interactions to characterize functional dynamics of the Hsp90 molecular chaperones and formulate a unifying theoretical framework that may adequately describe and rationalize molecular principles of Hsp90 dynamics and function.

## Results

We begin by providing a road-map through the manuscript and formulating main questions, computational experiments conducted and hypotheses tested. In the present work, we integrated all-atom MD simulations with modeling of principal correlated motions, the energy landscape analysis and structure-functional characterization of regulatory interactions to investigate functional dynamics and mechanisms of the Hsp90 molecular chaperones. The following specific objectives were pursued in this study: (a) to quantify molecular mechanisms by which functional dynamics and allosteric motions of Hsp90 may be connected with the probabilistic nature of the underlying energy landscape; (b) to identify and characterize functional hotspots shared by the Hsp90 chaperones that could coordinate functional dynamics and “conformational selection” of the functional states. We proposed that conformational dynamics and functional motions of Hsp90 may be described using the energy landscape models and principles of “conformational selection” (or “population-shift”) between preexisting conformational ensembles [99]–[101].

In the first section, we discussed the results of MD simulations performed for all structural forms of Hsp90. A comparative analysis of the conformational ensembles highlighted fundamental aspects of Hsp90 dynamics that may be exploited by the chaperone to accommodate functional requirements during the ATPase cycle. In the second section we characterized functional dynamics and principal collective motions of the molecular chaperone. This analysis demonstrated that a central evolutionary conserved characteristic of Hsp90 dynamics is the presence of long-range inter-domain motions that enable allosteric signaling. We concluded that allosteric interactions may provide a mechanism for structural adaptation of Hsp90 by shifting the dynamic equilibrium between the ensembles of functional states. A switch-based conformational change is a hallmark of complex biomolecular systems that function via allosteric signaling and are often regulated by a relatively small number of functional hot spots [128]. This mechanism may be characterized by coordinated collective motions where hinge regions could play a guiding role in changing the relative position of the rigid domain segments [102], [108], [109]. In the third section, we described a computational approach that identified and characterized a network of conserved functional motifs responsible for regulation of Hsp90 dynamics. In the subsequent section, by combining computational modeling and structure-functional analyses of regulatory interactions, we show that functional coupling of structurally stable and conformationally mobile elements in the regulatory motifs can enable allosteric signaling and recognition of co-chaperones and protein clients. The central finding of this study is that a relatively small number of functional motifs may be utilized by the chaperone machinery to act collectively as central regulators of Hsp90 dynamics and activity, including the inter-domain communications, control of ATP hydrolysis, co-chaperone binding and protein client recognition.

### Molecular Dynamics Simulations of the Hsp90 Molecular Chaperone

**Figure 1:**
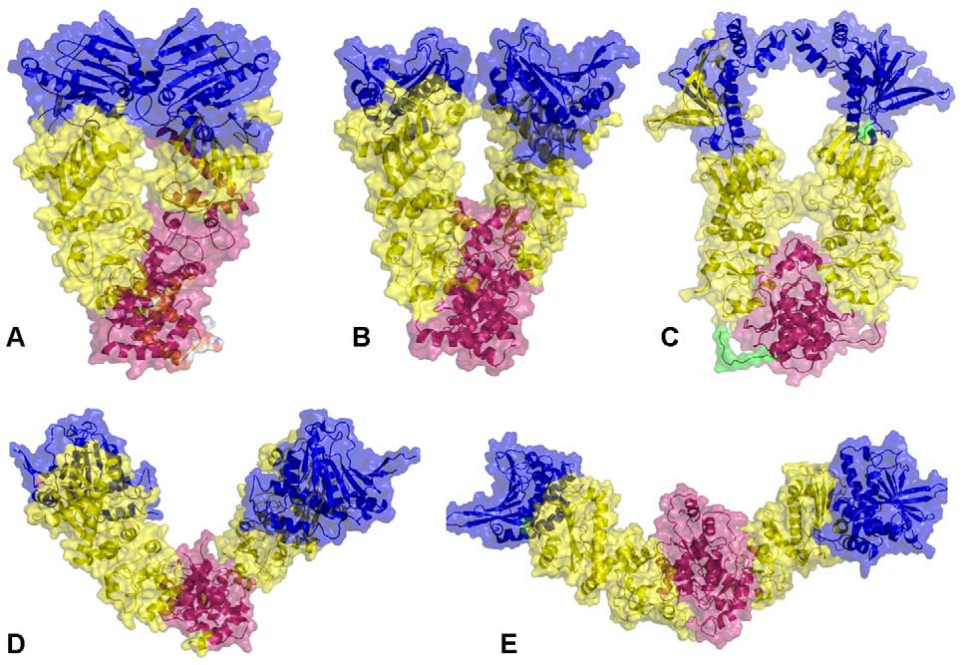
The Structures of the Full-Length Hsp90 Dimer. All available structures of the full-length Hsp90 dimer were employed in this study including : (**A**) A closed ATP-bound conformation of yeast Hsp90 (crystal structure) [54]; (**B**) A “V-shaped” conformation of the mammalian Grp94 homologue (crystal structure) [56]; (**C**) A semi-closed, ADP-bound form of the bacterial homologue HtpG (crystal structure) [55]; (**D**) An open Apo form of the bacterial homologue HtpG (crystal structure) [55]; and (**E**) An extended Apo HtpG conformation (solution structure from SAXS studies [61]. Each monomer in the yeast Hsp90 crystal structure is formed by the NTD (residues 2–215), M-domain (residues 262–526) and CTD (residues 527–676) (**A**). Each monomer of the Grp94 homologue is composed of residues 85–749. The disordered loops composed of residues 166–196, 286–329 and 396–407 were not included in the crystal structure [56]. Each monomer includes the NTD (residues 85–285), M-domain (residues 340–600) and CTD (residues 601–759) (**B**). Each monomer of the HtpG homologue [55] is divided into three domains, the NTD (residues 8–228), M-domain (residues 232–493), and CTD (residues 501–624) (**C–E**). The Pymol program was used for visualization of Hsp90 structures (The PyMOL Molecular Graphics System, Version 1.2r3pre, Schrödinger, and LLC). The NTD residues are shown in blue, the M-domain residues are in yellow-greenish and the CTD residues are in pink. The Hsp90 structures are depicted in ribbons overlayed with the surface representation at the 50% transparency according to PyMOL.

**Figure 2:**
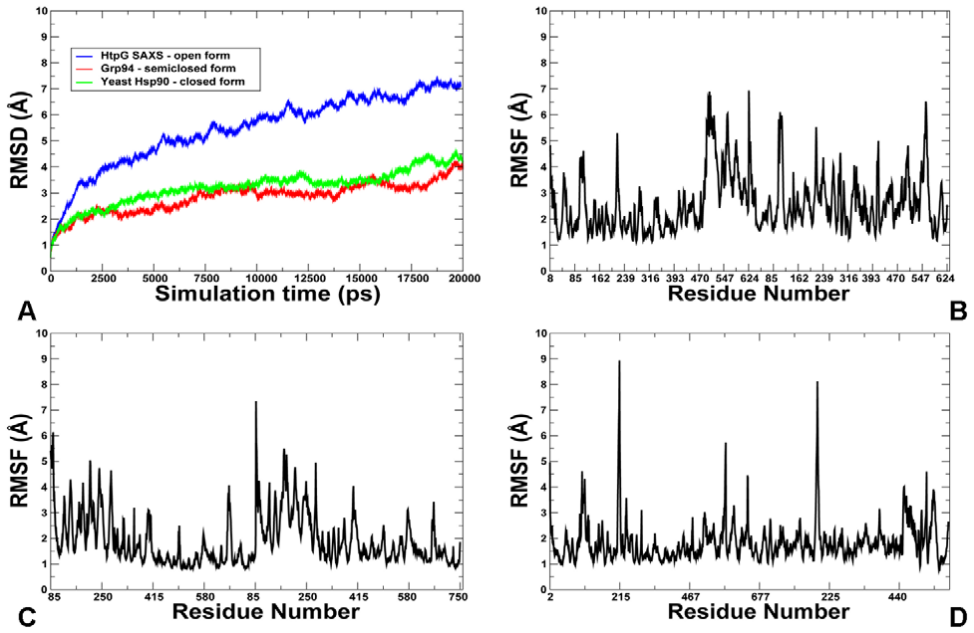
MD Simulations of the Hsp90 Structures. The results of all-atom 20 ns MD simulations are shown for three representative Hsp90 structures: a closed conformational state of yeast Hsp90 [54], a semi-closed conformation of the mammalian Grp94 homologue [56], and an open solution structure of the bacterial homologue HtpG [61]. (**A**) The RMSD fluctuations of the Cα atoms obtained from MD simulations of the bacterial homologue HtpG solution structure (in blue); mammalian Grp94 homologue (in red); and yeast Hsp90 (in green). The RMSF values of the Cα atoms obtained from MD simulations of the bacterial homologue HtpG solution structure (**B**), mammalian Grp94 (**C**), and yeast Hsp90 (**D**).

**Figure 3:**
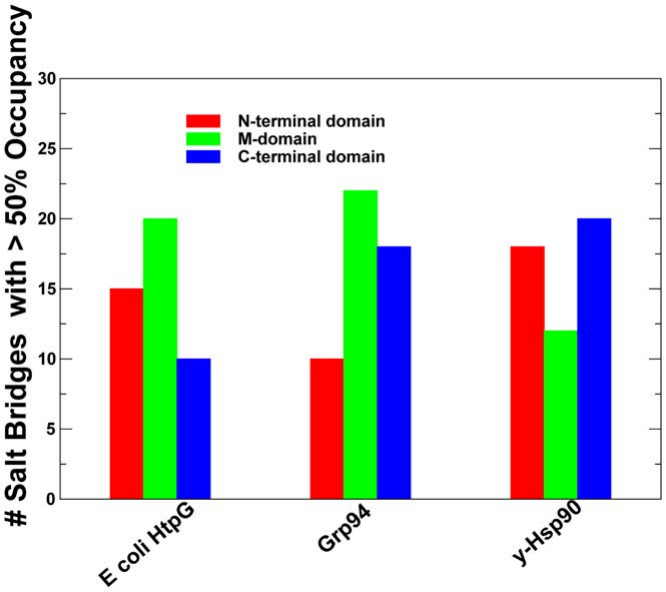
The Distribution of Salt Bridges in the Hsp90 Structures. The domain-based distribution of the high occupancy salt bridges derived from MD simulations of Hsp90 structures. The number of salt bridges are shown for the NTD (in red filled bars), M-domain (in green filled bars), and CTD (in blue filled bars).

The crystal structures of the full-length Hsp90 dimer from three different species revealed a similar domain architecture, yet significant differences in the global conformational flexibility and the relative domain orientation (**Figure 1**). To facilitate a comparative analysis of Hsp90 dynamics, we focused in this section on the results of all-atom MD simulations for three representative Hsp90 forms : a closed conformational state of yeast Hsp90 [54] (**Figure 1A**), a “twisted, V-shaped” conformation of the mammalian Grp94 homologue [56] (**Figure 1B**), and an extended Apo structure of the bacterial homologue HtpG obtained from SAXS experiments [61], [64] (**Figure 1E**). We first proceeded by monitoring the time dependent evolution of MD trajectories for the Hsp90 dimers using the root mean square deviation (RMSD) for all backbone atoms and the secondary structure elements (**Figure 2A**). The protein flexibility variations were computed from the root mean square fluctuation (RMSF) values of the backbone residues (**Figures 2 B–D**). A comparative analysis of conformational mobility indicated a greater degree of thermal fluctuations in the extended HtpG form, which was manifested in the larger RMSD values and a slower convergence of simulations (**Figure 2A**). The M-domain segments 319–334 and 336–354 have relatively small RMSF values and could be intrinsically stable in the extended HtpG form (**Figures 2 A, B**). According to HX-MS experiments [70], amide hydrogen protection and structural rigidity may increase upon ATP binding in the HtpG residues 2–19, 21–31, 90–98, 121–127, 192–206, 319–334, and 336–359. Despite a considerable residual flexibility, which is inherently present in the extended HtpG structure, structural integrity of secondary structure elements was largely preserved in all domains during simulations. The presence of diffuse and dense networks of stable salt bridges was most notable in the NTDs and the M-domains of HtpG (**Figures 3** **,** **4**). While the exposed and structurally separated NTDs were clearly prone to larger functional motions, stable networks of hydrogen bond interactions were seen in all three domains of each monomer, revealing a dense connectivity of both intra- and inter-domain salt bridges in the HtpG homologue. Consequently, the most stable salt bridges with the average occupancy exceeding 90% involved mostly the M-domain and the inter-domain pairs Asp309-Lys298, Asp308-Lys298, Glu448-Arg451, Glu471-Lys365, Asp462-Lys383, and Asp465-Lys383. The high occupancy hydrogen bonds were also present in the NTDs (Glu58-Arg142, Asp60-Arg172, and Asp41-Lys45) and in the CTDs (Glu569-Arg531, Glu545-Lys524, and Glu609-Arg605) (**Figures 3** **,** **4**).

**Figure 4:**
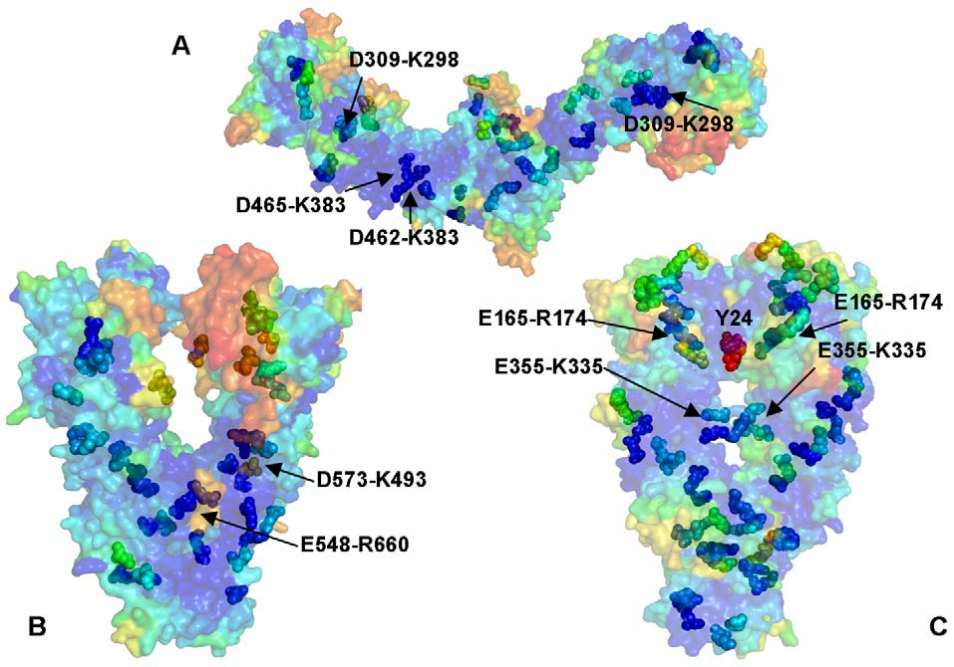
Hydrogen Bonding Networks in the Hsp90 Structures. Structural mapping of stable salt bridges obtained from MD simulations of the bacterial homologue HtpG solution structure (**A**); mammalian Grp94 homologue (**B**); and yeast Hsp90 (**C**). The Hsp90 conformations are shown in a surface-based representation colored (blue-to-red) according to the protein residue motilities (from more rigid-blue regions to more flexible-red regions). The salt bridge pairs are shown as spheres with the coloration reflecting the mobility of the respective residues forming these pairs. Structurally and evolutionary conserved high occupancy salt bridges are annotated and pointed to by arrows.

A markedly different equilibrium ensemble emerged from MD simulations of the Grp94 and yeast Hsp90 forms. The smaller RMSD values obtained for the semi-closed Grp94 and closed yeast Hsp90 structures (**Figure 2A**) reflected rather minor thermal fluctuations and a considerable structural stability of these functional states. MD simulations were generally stable within a fluctuation range of RMSD = 2 Å–3.5 Å and displayed a similar convergence to an equilibrium plateau after the first 3 ns. The trajectories remained mostly stable for the rest of the simulation period converging to the RMSD values of 3 Å–3.8 Å. Both trajectories paralleled except for the simulation period between 8 ns and 11 ns. Interestingly, simulations of the Grp94 structure proceeded in a fully stable regime after 4 ns and until about 14 ns, followed by a noticeable conformational change and increased fluctuations finally reaching the plateau at RMSD = 4 Å (**Figure 2A**). The RMSF values showed that the NTD regions were considerably more flexible in Grp94 than the M-domain and the CTDs (**Figure 2C**). The Grp94 mobile regions corresponded to the N-terminal flexible loops, the interface region between the M-domain and the CTD (residues 573–575) and the C-terminal loops (residues 652–682). The crystal structures of the Grp94 dimer in complexes with ATP and ADP are virtually identical, with the extensive contacts at the C-terminal interface (**Figure 4B**). The network of stable salt bridges in Grp94 reflected the dominant stabilizing interactions in the M-domain and CTDs. The high occupancy specific contacts included Glu548-Arg660, Glu564-Lys530 Asp573-Lys493, Asp584-Lys508 (**Figure S1**) that may serve as stabilizing “handlers” in assisting the central “strap” of hydrophobic residues (residues 755–765) to ensure structural integrity of the M-domain and CTD (**Figure 4B**). The key role of the M-domain and CTD in maintaining structural stability of Grp94 is fully consistent with the experimental data, wherein the truncation of the CTD residues was shown to result in a considerable loss of the hydrolysis activity [56]. The lack of the inter-domain specific interactions between Grp94-NTD and the M-domain may help to explain why ligand-induced conformational changes in Grp94 are limited only to the N-termini regions [51], [52], [56].

Consistent with our previous studies [75], MD simulations of yeast Hsp90 conformed to a “closed” dimeric conformation where both the NTDs and CTDs retained a tightly closed structural arrangement (**Figure 2D**). The “tense” structural form of the ATP-bound Hsp90 was evidenced from small thermal fluctuations observed in simulations. These results are fully consistent with the experimental data suggesting that the increase in structural rigidity and a globally more ordered state is a fundamental characteristic of the Hsp90 complex with ATP [75]. We observed a generally uniform distribution of salt bridges across domains with the tight clusters in the NTDs (Glu165-Arg174, Glu4-Lys86, Glu88-Lys102, Glu106-Lys48, Glu106-Lys102) and CTDs (Glu355-Lys335) holding the respective domains in the close contact (**Figure 4C**). These bridges tend to form an enclosed “ring” surrounding a critical Y24 residue that may contribute to the structural stability of the closed NTDs dimer arrangement [54], [75]. The dense network of both intra- and inter-domain salt bridge clusters in yeast Hsp90 may be an important stabilizing factor in securing the close structural arrangement of the monomers (**Figure 4C**). Importantly, structural rigidity of the closed Hsp90 form may coexist with conformational mobility of the linker regions at the inter-domain interfaces that are necessary to enable functional allosteric motions.

The conserved networks of hydrogen bond interactions preserved in different structural forms of Hsp90 are likely to be relevant in functional dynamics of the molecular chaperone. Despite vast structural differences the average number and domain-based distribution of high occupancy salt bridges was rather similar among Hsp90 structures (**Figures 3** **,** **4**). Another important finding was that the most stabilizing conserved salt bridges (>95% occupancy) were typically the interactions formed at the inter-domain interfaces, which may be an important structural requirement to enable the long-range inter-domain communication and efficient allosteric signaling.

### Functional Dynamics and Principal Collective Motions of the Hsp90 Molecular Chaperone

Conformational ensembles of the Hsp90 chaperone revealed significant differences in the global mobility of functional states. Crystallographic and solution-based studies of the bacterial homologue HtpG presented an ideal baseline for the initial comparison of computational and experimental data by revealing structural diversity of functional states, including a V-shaped apo-form and a semi-closed ADP-bound form of the bacterial homolog HtpG [55]; an extended solution structure of apo-HtpG [61], and a semi-closed state similar to the Grp94 crystal structure [56], [65]. According to the experimental studies, a highly mobile ADP-bound form of Hsp90 may be the most prominent single characteristic of the HtpG chaperone [70]. Another important experimental result is the evidence of anti-correlated motions between the C-terminal open and an N-terminal closed state [73].

These data indicated that structural adaptation of the Hsp90 chaperone may be regulated by the long-range inter-domain interactions. In this section, we combined analysis of the principal collective motions for all Hsp90 functional forms with the energy landscape model to test this hypothesis. The principal collective motions of the Hsp90 molecular chaperone were analyzed using principal component analysis (PCA) of MD trajectories. The structural distribution of the protein mobility and the cross-correlation maps of protein residue fluctuations were computed along the dominant principal component (PC) modes. This analysis identified the shapes of the low frequency modes and the protein regions subjected to correlated and/or anti-correlated motions along the selected low frequency modes.

**Figure 5:**
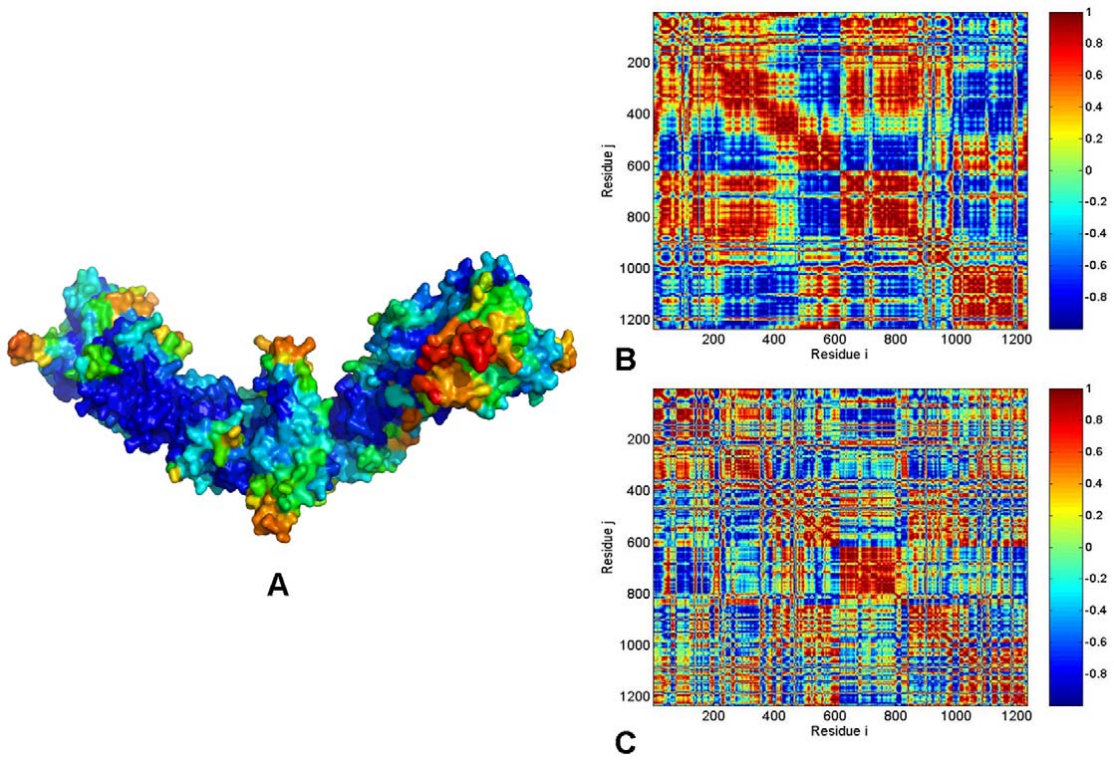
PCA of Functional Motions in the HtpG Chaperone. (**A**) Structural distribution of the HtpG mobility along the dominant, lowest frequency mode obtained from all-atom MD simulations. A surface-based protein representation is employed, colored (blue-to-red) according to the protein residue motilities (from more rigid-blue regions to more flexible-red regions). The cross-correlation covariance matrix of residue fluctuations was computed along the first slowest PC mode (**B**) and the second slowest PC mode (**C**). The matrix was calculated based on MD trajectories of the bacterial homologue HtpG in the extended solution conformation [61]. The essential directions of correlated motions during dynamics were then calculated by diagonalizing the covariance matrix *C_ij_*. Cross-correlations of residue-based fluctuations vary between +1 (fully correlated motion; fluctuation vectors in the same direction, colored in dark red) and −1 (fully anti-correlated motions; fluctuation vectors in the same direction, colored in dark blue). Here, the values above 0.5 are colored in dark red and the lower bound in the color bar indicates the value of the most anti-correlated pairs. Each monomer of the solution structure of the HtpG homologue [55] is divided into three domains with the following residue numbering NTD (residues 8–228), M-domain (residues 232–493), and CTD (residues 501–624). In the cross-correlation matrix, the domain annotation in the monomer 1 is the following: NTD (residues 1–221). M-domain (residues 225–486), CTD (residues 496–617). The monomer 1 is composed of residues 1–617; the monomer 2 corresponds to residues 618–1234.

Sstructural mapping of the HtpG mobility projected along the low frequency modes revealed a heterogeneous and diffuse network of structurally stable regions distributed across all domains of the extended HtpG form (**Figure 5A**). Conformationally flexible segments corresponded to the “tips” of the HtpG-CTDs and solvent-exposed regions of the HtpG-NTDs. The cross-correlation matrix of residue fluctuations driven by the two dominant PC modes displayed a considerable degree of positive cross-correlations that extended beyond the intra-domain regions, indicating the existence of long-range interactions and coordinated domain movements (**Figure 5 B,C**). The protein mobility distribution of the HtpG residues averaged over the slowest two modes reflected a considerable structural rigidity of the M-domains. A greater structural mobility of the NTDs and CTDs acting as rigid bodies may facilitate opening-closing movements of the HtpG chaperone. We determined that collective movements of HtpG may be largely determined by positive cross-correlations between the NTD (residues 8–228) and M-domain (residues 232–493). Importantly, these correlated motions may coexist with the concerted anti-correlated motions between the NTD and CTD (residues 501–624) (**Figure 5 B, C**). These results suggested that allosteric inter-domain interactions may control global motions and regulate the molecular chaperone machinery. These results are among central findings of our analysis and are consistent with the recently discovered coordinated kinetics of the CTD and NTD that confirmed the existence of anti-correlated motions between the NTDs and CTDs [73].

**Figure 6:**
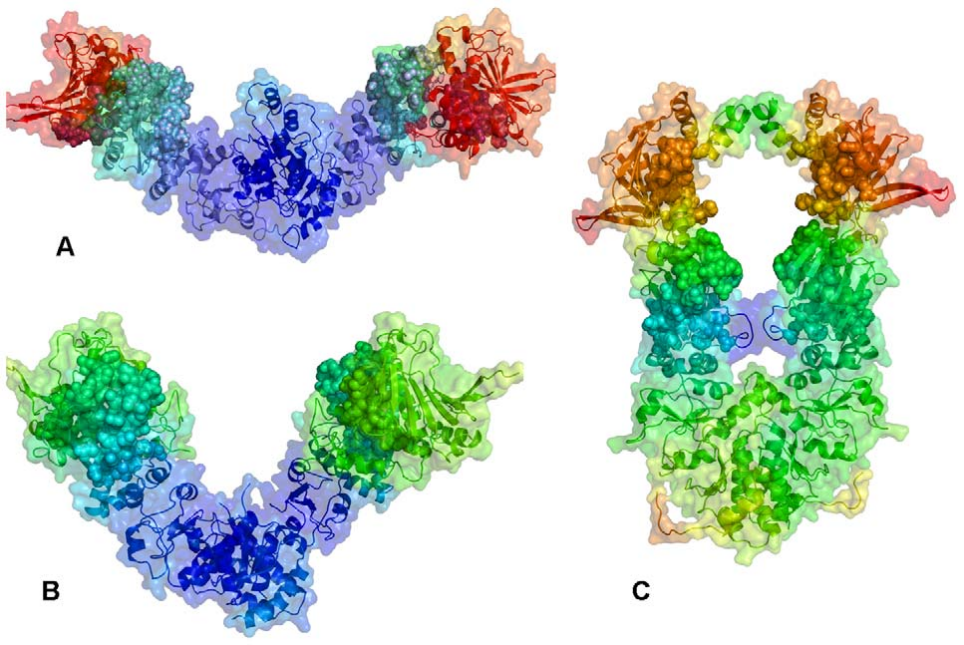
Protein Mobility of the Functional Regions in the HtpG Structures. The protein mobility of the HtpG structures : (**A**) extended apo-HtpG solution structure; (**B**) V-shaped apo-HtpG; and (**C**) ADP-bound, compact HtpG form. For comparison with the experimental data, the following protein segments monitored in HX-MS experiments [70] were mapped onto the HtpG structures: residues 121–127 (NTD), 192–206 (NTD), 319–334 (M-domain), and 336–359 (M-domain). The Hsp90 structures are depicted in ribbons overlayed with the surface representation at the 50% transparency according to PyMOL. A surface-based representation colored (blue-to-red) according to the protein residue motilities (from more rigid-blue regions to more flexible-red regions). The functional protein segments are shown as spheres with the coloration reflecting changes in their mobility among different structural forms of HtpG.

Conformational mobility in functionally important regions can be often modulated by nucleotide binding. The last helix of the NTD (residues 192–200), the catalytic loop in the M-domain (residues 332–342) and the adjacent H10 helix (residues 343–366) are among functional regions that become protected upon ATP binding [70]. According to our data, structural stability of these functional regions was preserved in both solution structure (**Figure 5**) and crystal structure of apo-HtpG (**Figure 6**) where the respective residues corresponded to a more rigid part of the mobility spectrum. Another important finding was the greater structural integrity of the extended solution HtpG structure (**Figure 6A**) and a V-shaped crystal structure of the apo-HtpG (**Figure 6B**) as compared to a more compact, yet highly flexible ADP-form of HtpG (**Figure 6C**). A considerable mobility of the ADP-bound HtpG was detected in all domains reflecting the intrinsic flexibility present in this otherwise fairly compact structural form. Our computational analysis suggested that the intrinsic flexibility and transient nature of the ADP-bound HtpG conformation may be determined by the weakening of the inter-domain interactions and a reduction in the long-range communication within each of the monomers. These results are in excellent agreement with the HX-MS and fluorescence spectroscopy studies of the HtpG dynamics [70]. SAXS studies have also unveiled that the ADP-bound HtpG structure could not be detected as a stable HtpG intermediate in solution and this chaperone conformation may be only transiently present in kinetic transitions [61], [70]. To summarize, the presence of anti-correlated motions between the NTD and CTD in the open apo-HtpG form, and a highly mobile ADP-bound form are the two fundamental characteristics of the HtpG chaperone dynamics [70].

**Figure 7:**
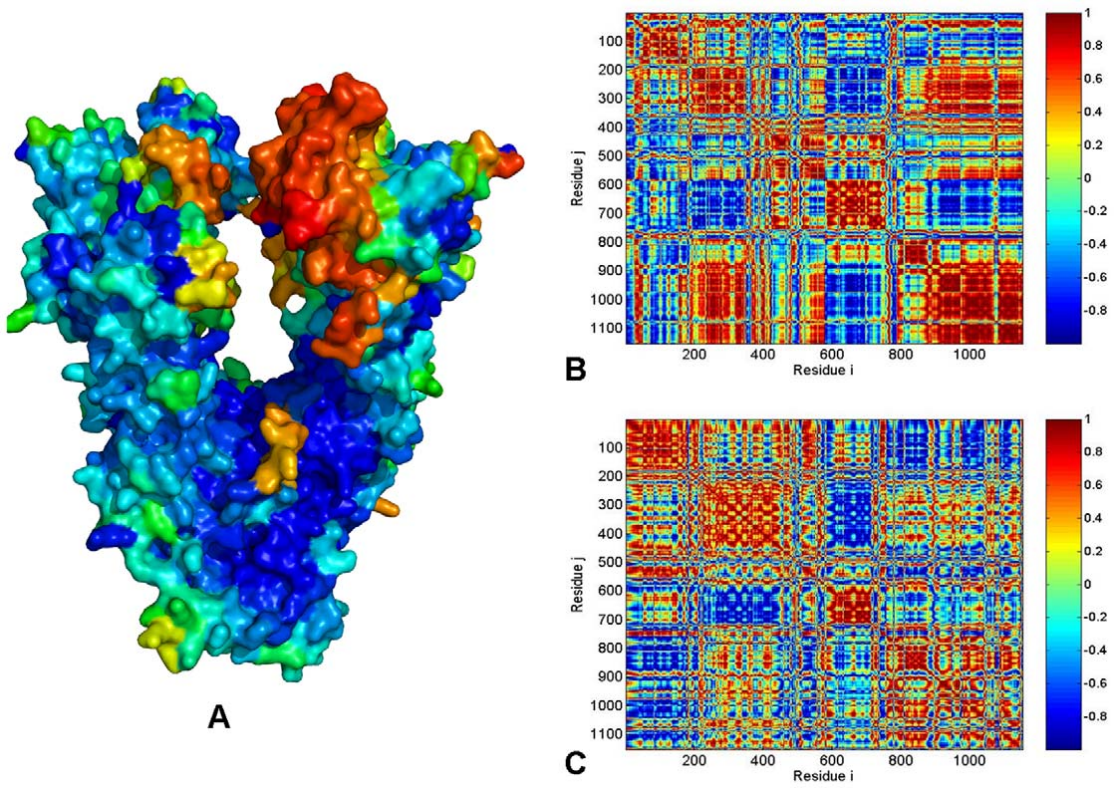
PCA of Functional Motions in the Grp94 Chaperone. (**A**) Structural distribution of the Grp94 mobility along the dominant, lowest frequency mode obtained from all-atom MD simulations. The cross-correlation covariance matrix of residue fluctuations was computed along the first slowest PC mode (**B**) and the second slowest PC mode (**C**). A surface-based representation colored (blue-to-red) according to the protein residue motilities (from more rigid-blue regions to more flexible-red regions). Each monomer of the Grp94 homologue [56] is divided into three domains with the following residue numbering in the crystal structure: NTD (residues 85–285), M-domain (residues 340–600) and CTD (residues 601–759). The disordered loops (residues 166–196, 286–329 and 396–407) not included in the crystal structure [56] were removed in the computation of the cross-correlation matrix. As a result, the domain/residue annotation in the monomer 1 is the following: NTD (residues 1–180), M-domain (residues 181–429), and CTD (residues 430–578). The monomer 1 is composed of residues 1–578; the monomer 2 corresponds to residues 579–1154.

The full-length mammalian Grp94 in complex with either ATP or ADP has a semi-open, V-shaped conformation of the dimer [56]. Structural mapping of the Grp94 mobility (**Figure 7A**) and the residue-based cross-correlation maps along the two slowest PC modes (**Figure 7B, C**) revealed positive cross-correlations between the M-domain and CTD of the same monomer as well as between the monomers. This analysis clearly indicated that the Grp94-NTD could be more flexible than the other domains, and thermal fluctuations of the Grp94-NTD may be decoupled from highly coordinated motions of the M-domain and CTD. This central feature of the functional dynamics in Grp94 may impair the formation of productive dimerization interactions between the two NTDs that are necessary for ATP hydrolysis. The computational findings could thus partly explain the experimental data [56], according to which conformational changes in Grp94 may be limited only to the NTDs. The lower catalytic rate of Grp94 as compared to yeast Hsp90 [56] may be also associated with the lack of efficient long-range communication for Grp94-NTDs. This may ultimately hamper the formation of the catalytically productive structure.

**Figure 8:**
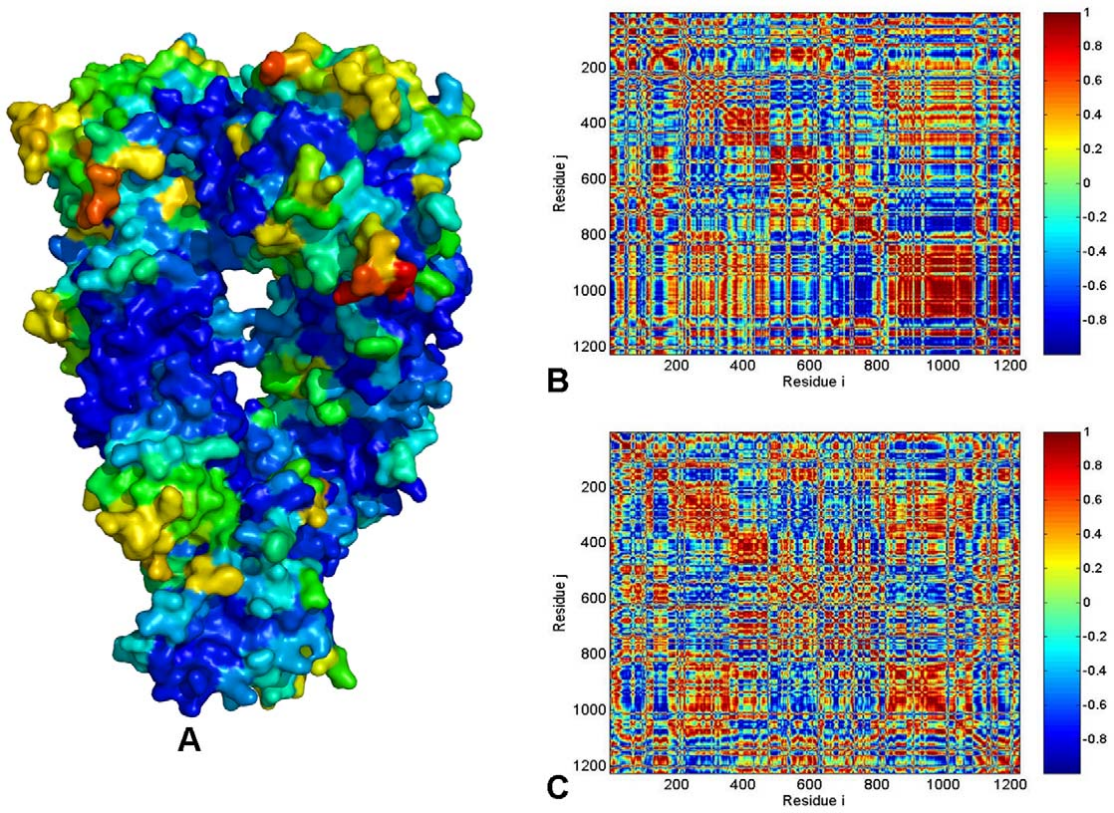
PCA of Functional Motions in the Yeast Hsp90 Chaperone. (**A**) Structural distribution of yeast Hsp90 mobility along the dominant, lowest frequency mode obtained from all-atom MD simulations. The cross-correlation covariance matrix of residue fluctuations was computed along the first slowest PC mode (**B**) and the second slowest PC mode (**C**). A surface-based protein representation is employed, colored (blue-to-red) according to the protein residue motilities (from more rigid-blue regions to more flexible-red regions). In the cross-correlation matrix, the domain annotation in the monomer 1 is the following: NTD (residues 2–215), M-domain (residues 262–526) and CTD (residues 527–676). The monomer 1 consists of residues 2–676; the monomer 2 corresponds to residues 677–1351.

The ATP-bound yeast Hsp90 is characterized by structurally constrained conformation, stabilized by a dense network of both intra-monomer and inter-monomer interactions (**Figure 8A**). The cross-correlation matrix of residue fluctuations revealed positive correlations between the NTD and CTD residues within each protomer (**Figure 8 B, C**), which may be considered as an important intrinsic feature of the closed Hsp90 form. The functional role of this interaction network is likely to stabilize the closed “tense” state of the molecular chaperone and enable a globally concerted dynamics of the catalytically competent Hsp90 conformation. Functional coupling between the two protomers was seen in the positive correlations of allosterically communicating NTD and CTD regions within the monomers as well as between the M-domains of two monomers (**Figures 8**). In agreement with the experimental data [73], our analysis also pointed to the presence of anti-correlated coupling between the NTD of one monomer and the CTD of the other monomer. The correlation analysis of the Hsp90 dimer in different species (**Figures 5** **,** **6** **,** **7** **,** **8**) revealed an appreciable degree of correlation between NTD and CTD motions in HtpG and yeast Hsp90. The functional impairment of the Grp94 form to effectively coordinate ATPase-based functional changes [56] may be directly linked to the observed poor inter-domain interactions of Grp94-NTDs, suggesting that allosteric coupling may be fundamental to Hsp90 activity. Hence, analysis of principal collective motions demonstrated that a central evolutionary conserved characteristic of Hsp90 dynamics is the presence of long-range inter-domain motions that could enable allosteric signaling.

These findings are in accordance with insights by Wolynes and coworkers [129], [130] who have recently advanced the energy landscape theory of allosteric regulation. According to this theory, proteins may have evolved to ensure relatively unfrustrated interactions between amino acids and long-range cooperativity. Our results suggested that the energy landscapes of the Hsp90 chaperone may have acquired the ability for regulation via cooperative allosteric changes. The presence of allosteric interaction networks and robust inter-domain communications in the functional Hsp90 forms is fundamental characteristic of relatively unfrustrated energy landscape. A certain degree of local frustration is always present in an otherwise largely unfrustrated protein structure and may have arisen from evolutionary requirements to adapt protein dynamics for specific functions [129], [130]. In contrast, the energy landscape of the Grp94 form may be more frustrated, reflecting the lack of cooperativity between the NTDs and other domains within the same protomer and between the two different protomers.

Within the energy landscape framework, long-range cooperativity of allosteric interactions during allosteric transitions may favor a combination of the population-shift and induced-fit mechanisms [130]. From a thermodynamic perspective, the conformational selection model and induced fit can be considered as complementary models, as due to thermal fluctuations there is always are some degree of the induced fit on the atomic scale of molecular interactions. Based on these arguments and our results, we suggested that allosteric signaling in the Hsp90 chaperone may involve a mechanism of “conformational selection” and “population-shift” between the ensembles of functional states seen in the crystal and solution structures.

### Universal Regulatory Motifs of Hsp90 Dynamics and Allosteric Signaling

Allosteric signaling is often enabled by a relatively small number of evolutionary conserved and sparsely connected regulatory hotspots. In this section, we describe a computational approach to identify and characterize a network of conserved functional motifs responsible for regulation of Hsp90 dynamics. We propose that functional motifs coordinating functional dynamics and collective motions of the Hsp90 chaperone may serve as central regulators of Hsp90 activity. Structurally rigid and conformationally flexible residues may often occupy proximal positions to the hinge sites, since the regions of high and low structural stabilities could participate in binding sites [107]. We propose that functional sites of Hsp90 involved in allosteric signaling, catalysis and binding may be strategically positioned near key anchoring regions and inter-domain interfaces to control global movements of the molecular chaperone. By integrating computational modeling with structure-functional analyses of regulatory interactions, we show that that functional coupling of structurally stable and conformationally mobile elements in the regulatory motifs can enable allosteric signaling and recognition of co-chaperones and protein clients.

We focus on the low-frequency functional motions represented by the normalized mean square fluctuation (NMSF) of the Hsp90 chaperone residues, along the two lowest frequency modes. These profiles can reflect salient features of the intrinsic protein dynamics [105]–[107]. In the distribution of residue fluctuations along the slowest modes the maxima are typically considered as more flexible recognition sites and the minima as globally anchored regions which may act as hinge residues driving global conformational changes. The analysis of Hsp90 functional dynamics revealed a considerable heterogeneity of structurally rigid and flexible residues forming overlapping clusters in the interaction networks. Consequently, we expanded the previously developed model by focusing also on the “inflection points” of the fluctuation profiles along the slow modes. In these regions the sign of the NMSF function curvature (i.e. the concavity) can change, representing “turning points” in the function behavior, reflecting sharp transitions from maxima to minima. In this computational model, we assumed that the residues that display cross-correlation of their thermal fluctuations and that are stationary points of the NMSF function along the slow PC modes may determine long-range collective motions of Hsp90 functional dynamics. While the residues corresponding to the NMSF minima may communicate efficiently, the higher mobility residues exhibiting a concerted change in their fluctuations may also contribute to the allosteric interaction networks of the Hsp90 chaperone.

### The Inter-domain Charged Linker and Catalytic M-domain Motif Can Regulate Hsp90 Allosteric Signaling

**Figure 9:**
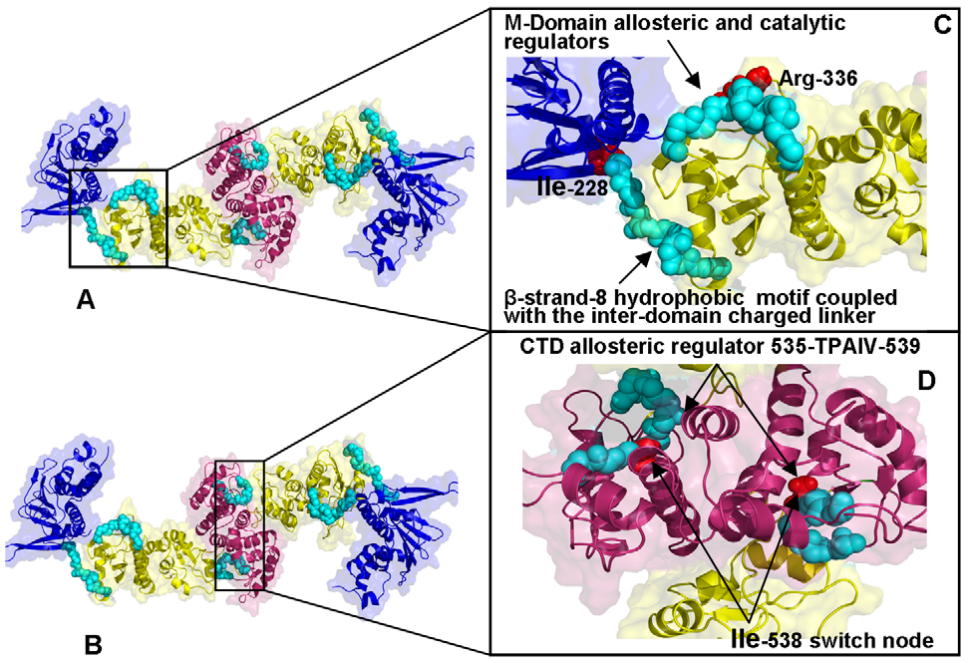
Structure-Functional Analysis of Allosteric Regulators in the HtpG Homologue. (**A, B**) Structural mapping and overview of major functional regions corresponding to the stationary points in the NMSF profiles. These residues may be involved in coordinating conformational dynamics. The Pymol program was used for visualization of Hsp90 structures. The NTD residues are shown in blue, the M-domain residues are in yellow-greenish and the CTD residues are in pink. The Hsp90 structures are in ribbons overlayed with the surface representation at the 50% transparency according to PyMOL. (**C**) A close-up view of the β-strand-8 hydrophobic motif, the inter-domain charged linker and the M-Domain allosteric regulatory region. The functional regions are shown in cyan spheres depicting only the main chain amino acids. The catalytic residue Arg-336 is highlighted in red spheres. (**D**) A close-up view of the CTD functional regulatory region (residues 535-TPAIV-539). The functional region is shown in cyan spheres and the critical switch node residue Ile-538 is shown in red spheres.

**Figure 10:**
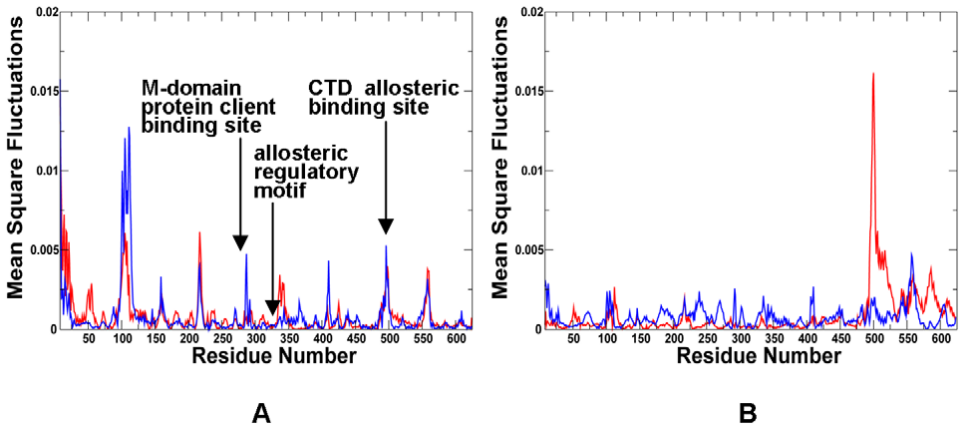
The Residue-Based Fluctuation Profiles of the HtpG Homologue. The NMSF residue profiles derived from PCA of all-atom MD simulations along the lowest frequency mode (**A**) and the second lowest mode (**B**). The profiles are shown for the monomer 1(in blue) and monomer 2 (in red). Each monomer of the HtpG homologue [55] is divided into three domains, the NTD (residues 8–228), M-domain (residues 232–493), and CTD (residues 501–624). The conserved regulatory and recognition sites are annotated and pointed to by arrows.

According to this analysis, the NMSF profiles along the two slowest modes of HtpG motions confirmed the presence of anti-correlated motions between the NTD and CTD in the open apo-HtpG form (**Figures 9** **,** **10**). The fluctuation profile along the slowest mode displayed a noticeable peak in the NTD region (residues 90–125) coexisting with more suppressed fluctuations in the CTD, indicative of large amplitude opening-closing motions of the NTDs. Conversely, the profile along the second slowest mode (**Figure 10B**) exhibited a significant peak in the CTD region (residues 500–550) whereas the NTDs fluctuations were rather minor. As a result, principal collective motions in HtpG may involve opening of the CTDs with the NTD regions remaining in their stable positions. The local minima and inflection points of the HtpG fluctuation profiles along slow motion modes pointed to the inter-domain region (residues 225–240) and functional cluster in the M-domain (residues 334–344) (**Figure 10**). According to our hypothesis, these motifs may be involved in regulating the inter-domain interactions. The first segment 227-INKAQALWTRNK-238 corresponded to the hydrophobic motif from the β-strand-N8 containing the evolutionary conserved Ile-227 residue (a local minimum of the NMSF profile). The adjacent linker 228-NKAQALWTRNK-238 is situated between the NTD and M-domain (**Figures 9** **,** **10**) [71]. According to our model, the residues from this functional motif correspond to the stationary points of the fluctuation profile (**Figure 10**) and hence may be involved in the inter-domain communication and regulation of principal collective motions. It is reasonable to assume that mutations in these functional hotspots may weaken the inter-domain communication and compromise allosteric signaling in the chaperone. Indeed, the charged linker residues were implicated in mediating conformational changes needed for the ATP hydrolysis [71], [72]. According to these experimental findings the hydrophobic residue Ile-227 (corresponding to Ile-218 in human Hsp90 and Ile-205 in yeast Hsp90) may an important regulatory node of the chaperone activity. HX-MS and trypsin sensitivity analyses have demonstrated that alanine mutations of this regulatory residue may alter the chaperone activity by shifting Hsp90 conformational equilibrium to a more open state [71], [72].

Interestingly, this charged linker in Grp94 (residues 287–327) was deleted in the protein construct and replaced by four glycine residues in the crystal structures [56]. This region in the crystal structure was replaced by an internal loop (269-KKYSQFINFP-278) anchored by the interactions between the antiparallel β-strand-N8 and β-strand-N9 [56]. As a result of the protein construct design, the charged region is no longer a flexible linker connecting the NTD and M-domain, but rather an integral part of the Grp94-NTD [56]. According to our model, the absence of the functional linker connecting NTD and the M-domain in Grp94 may be responsible for the weakening of the inter-domain interactions. Indeed, analysis of the principal collective motions indicated a partial decoupling of the Grp94-NTD motions from the coordinated functional dynamics of the M-domain and CTDs (**Figure 7**). This may impair the ability of the Grp94 chaperone to effectively coordinate ATPase-based functional changes. Hence, allosteric machinery of Grp94 may not be “sufficiently equipped” to initiate large conformational changes associated with the ATPase cycle.

In the framework of the energy landscape model, this inter-domain region may facilitate cooperative interactions and efficient allosteric communication networks in HtpG. The alterations in this region observed in Grp94 may introduce a degree of inter-domain frustration that would detrimentally affect allosteric interaction networks. These findings could rationalize several important biochemical experiments [51], [52], [56], [65] which demonstrated that conformational dynamics of Grp94-NTD may be independent from functional motions in the M-domain and CTDs. Moreover, the nucleotide binding alone is not sufficient to cause conformational changes in Grp94 that are compatible with the efficient ATP hydrolysis [56]. As a result, co-chaperone and protein client binding may be needed to assist Grp94 in fulfilling the chaperone function.

**Figure 11:**
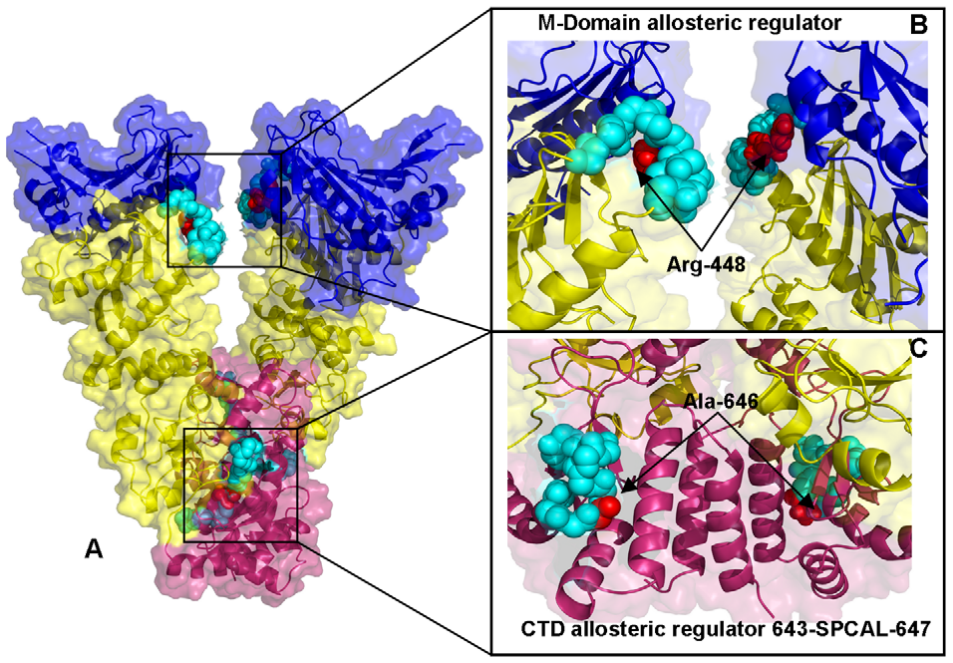
Structure-Functional Analysis of Allosteric Regulators in the Grp94 Chaperone. (**A**) Structural mapping and overview of the functional regions corresponding to the stationary points in the NMSF profiles. The Pymol program was used for visualization of Hsp90 structures. The NTD residues are shown in blue, the M-domain residues are in yellow-greenish and the CTD residues are in pink. The Hsp90 structures are in ribbons overlayed with the surface representation at the 50% transparency according to PyMOL. (**B**) A close-up view of the M-domain regulatory motif (residues 444–454 LNVSRETLQQ from the M-domain). The functional motif is shown in cyan spheres depicting only the main chain amino acids. The catalytic residue Arg-448 is highlighted in red spheres. (**C**) A close-up view of the CTD functional regulatory region (residues 643-SPC**A**L-647). The functional region is shown in cyan spheres and the critical switch node residue Ala-646 is shown in red spheres.

Another segment suggested by our analysis as a potential regulator of collective motions included conserved residues 332-LNVS**R**EILQD-342 in HtpG (**Figures 9** **,** **10**) and the respective region 444-LNVS**R**ETLQQ-454 in Grp94 (**Figures 11** **,** **12**). These motifs include the M-domain catalytic loop with a critical for ATP hydrolysis Arg-336 in HtpG and Arg-448 in Grp94. The catalytic loop and Arg-336 are both highly conserved and are essential for hydrolysis by Hsp90 [40]. According to our data, structural stability of this region was preserved in solution and crystal structures of apo-HtpG as well as in a highly mobile ADP-bound HtpG form. The covariance map of residue fluctuations revealed positive correlations of this region with the M-domain and CTD residues in both HtpG and Grp94, suggesting a likely involvement of this region in the inter-domain communication network (**Figures 5** **,** **6** **,** **7**). The slow-mode NMSF profile of yeast Hsp90 (**Figures 13** **,** **14**) signaled similar changes in the concavity of the fluctuation profile for residues 375–385 of the M-domain corresponding to the functional loop containing catalytic residue Arg-380 and connected to the structurally stable three-helix bundle. Hence, this functional motif in the M-domain may be involved in both allosteric coupling and ATP hydrolysis. Hence, the NTD β-strand-N8 hydrophobic motif, the inter-domain charged linker and the M-domain catalytic region motif may coordinate principal chaperone motions and allosteric conformational changes. The dual role of this regulatory motif in moderating principal collective motions of Hsp90 and ATP hydrolysis is an important finding of our study. These results support a long-standing assertion that allosteric regulation and catalysis may have emerged via common evolutionary route [**97**].

**Figure 12:**
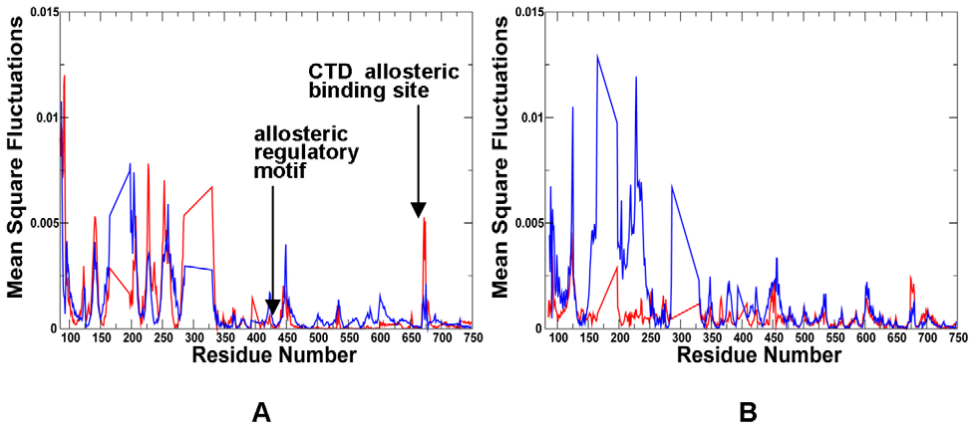
The Residue-Based Fluctuation Profiles of the Grp94 Chaperone. The NMSF residue profiles derived from PCA of all-atom MD simulations along the lowest frequency mode (**A**) and the second lowest mode (**B**). The profiles are shown for the monomer 1(in blue) and monomer 2 (in red). Each monomer includes the NTD (residues 85–285), M-domain (residues 340–600) and CTD (residues 601–759). The disordered loops composed of residues 166–196, 286–329 and 396–407 were not included in the crystal structure [56]. The conserved regulatory and recognition sites are annotated and pointed to by arrows.

**Figure 13:**
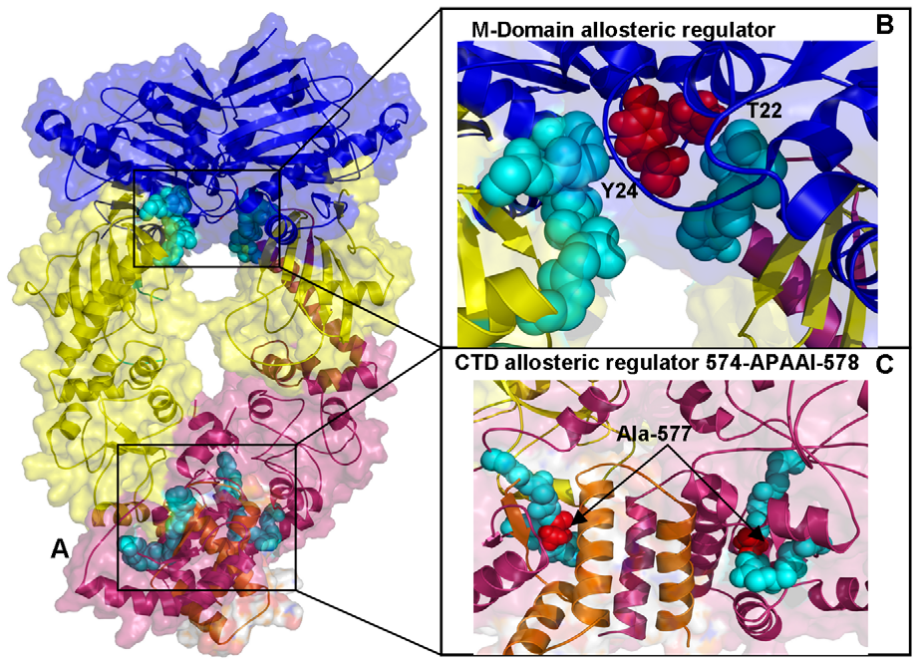
Structure-Functional Analysis of Allosteric Regulators in the Yeast Hsp90 Chaperone. (**A**) Structural mapping and overview of the functional regions corresponding to the stationary points in the NMSF profiles. The Pymol program was used for visualization of Hsp90 structures. The NTD residues are shown in blue, the M-domain residues are in yellow-greenish and the CTD residues are in pink. The Hsp90 structures are depicted in ribbons overlayed with the surface representation at the 50% transparency according to PyMOL. (**B**) A detailed close-up view of the functional region (residues 375–385 in the M-domain). The functional motif is shown in cyan spheres depicting only the main chain amino acids. The functionally important residues T22 and Y24 are highlighted in red spheres. (**C**) A detailed close-up view of the functional region (residues 570–580 from the CTD). The functional region is shown in cyan spheres and the critical switch node residue Ala-577 is shown in red spheres.

**Figure 14:**
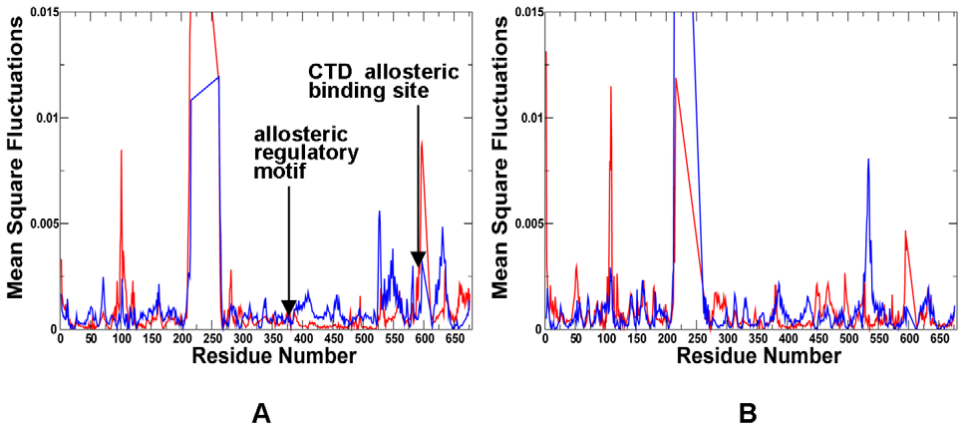
The Residue-Based Fluctuation Profiles of the Yeast Hsp90 Chaperone. The NMSF residue profiles derived from PCA of all-atom MD simulations along the lowest frequency mode (**A**) and the second lowest mode (**B**). The profiles are shown for the monomer 1(in blue) and monomer 2 (in red). Each monomer in the yeast Hsp90 crystal structure is formed by the NTD (residues 2–215), M-domain (residues 262–526) and CTD (residues 527–676). The conserved regulatory and recognition sites are annotated and pointed to by arrows.

### Functional Coupling of Regulatory Motifs Can Enable Allosteric Signaling and Binding with Protein Clients

The fluctuation profiles also revealed a consistent pattern of local maxima corresponding to more flexible recognition sites. According to our model, these sites may be involved in assisting global conformational rearrangements of Hsp90 and protein client recognition.

All these regions are strategically situated on the protein surface, at the inter-domain interfaces and at the tips of the NTDs and CTDs. In particular, these regions in HtpG included residues 215-EKD-21, 286-WDM-288, 410-SAQT-413, 491-EKLADE-496, and 556-AAGQK-561 (**Figures 9** **,** **10**). The detected recognition motif 286-WDM-288 belongs to the larger projecting loop 284-APWDMWNRDHKHG-296 in the M-domain of HtpG. This evolutionary conserved region (327-APFDLFESKKKKN-339) in yeast Hsp90 is believed to be involved in recognition of protein clients. Another smaller peak in the profile corresponds to the W257 residue in HtpG (**Figure 10**) and W300 in yeast Hsp90) (**Figure 14**) that is situated on the same face as the projecting loop and is also thought to be involved in recognition with protein kinases. The inner face of this patch involves the side chains of W286, M288, and W289 in HtpG (F329, L331, and F332 in yeast) which form the solvent-exposed tip of the amphipathic structure, with one hydrophobic side and one positively charged side. These residues may be involved in protein binding, since mutations in this region can impair chaperone function without loss of ATPase activity, suggesting detrimental changes in the client interactions [40]. The potential involvement of this M-domain loop in recognition of protein kinase clients was also supported in yeast two-hybrid experiments of Hsp90 binding with Akt kinase [131]. By identifying universal functional motifs involved in regulation of Hsp90 activity, these findings may clarify dynamics aspects of Hsp90 binding with co-chaperones and protein clients. In particular, our results may rationalize the insights from biochemical studies of Hsp90 binding with co-chaperone Cdc37, which is recruited by Hsp90 to recognize protein kinase clients [12]. Biochemical studies have indicated that Cdc37 binding to the Hsp90 dimer would cause structural rearrangements between the NTD and MD, which are arguably necessary during the client-loading phase of the chaperone cycle [12], [57], [59]. Additionally, HX-MS experiments have revealed considerable changes in the Hsp90 protection in the presence of Cdc37 indicative of a conformational change at the interface between NTD and the M-domain, and involving the catalytic loop [66]. In an excellent agreement with these experiments, our results demonstrated that allosteric communications in Hsp90 may be regulated by the functional loop connected to the 3-helix bundle and transmitted through interactions between α9-β8 in the NTD and β11 in the M-domain. The central involvement of the M-domain catalytic loop in allosteric signaling is further supported by the experiments showing that mutations in this region may dramatically affect Hsp90 activity [132], [133]. In particular, Hsp90 interactions with Cdc37 may be attenuated by mutations of T22 and Y24 residues in the NTD of yeast Hsp90 [**133**]. The catalytically competent closed structure in yeast Hsp90 is stabilized by a network of hydrophobic interactions between the catalytic loop residues and the α-helix-1 residues in NTD including T22 and Y24 (**Figure 13B**). Mutations of T22 and Y24 to either alanine or glutamic acid could disrupt Hsp90 association with co-chaperone Cdc37 and markedly reduce Hsp90 ATPase activity [132], [133]. Interestingly, mutations that may disfavor the ATP-induced conformational switch in the Hsp90 chaperone cycle (T101I) could substantially reinforce Hsp90-Cdc37 interactions, whereas a mutation favoring this conformational switch (T22I) would prohibit Cdc37 binding [134].

Hence, allosterically regulated conformational equilibrium of Hsp90 and co-chaperone binding are closely associated and may be modulated via the stochastic nature of the underlying energy landscape. Based on our findings and agreement with structure-functional experiments [135]–[138], we suggest that Hsp90-Cdc37 binding may be enabled by coordinated involvement of the inter-domain charged linker and the catalytic functional loop from the M-domain (**Figure S2**). When Hsp90 dimer forms an initial complex with two Cdc37 molecules [12], [57], these projecting loops may act as functional “arms” of the chaperone machinery to recognize and accommodate co-chaperone Cdc37 (**Figure S2**). According to our mechanistic model, the binding sites of these motifs may recognize the “incoming” co-chaperone molecules, and the Cdc37 dimer would then bind the two linker regions. The regulatory elements of these motifs may then “pass” the binding signal information via allosteric communication pathways and trigger switching of the thermodynamic equilibrium towards a more open and flexible ADP-bound state of Hsp90 suitable for recognition of protein kinase clients.

Our results also highlighted the relevance of the 491-EKLADE-496 CTD motif as a potential recognition site in HtpG (**Figures 9C** **,** **10**). Recent studies have demonstrated that the Hsp90 CTD is important for dimerization of the chaperone and contains a second nucleotide binding site [80]–[82]. Indeed, this motif is a part of the previously discovered allosteric binding site targeted by C-terminal inhibitors such as novobiocin [94], [95]. Biochemically guided modeling based on the bacterial homologue HtpG has recently revealed a putative binding mode of novobiocin inhibitor [96]. This recognition motif is situated around the functional region 535-TPA**I**V-539 containing a pivotal switch point Ile-538 in HtpG (**Figures 9C**). Mutation of this critical residue could alter chaperone activity, and disrupt inter-domain connectivity in Hsp90 [71], [72]. The CTD regulatory motif is evolutionary conserved and is formed by residues 643-SPC**A**L-647 in Grp94 (**Figures 11C**) and 574-APA**A**I-578 in yeast Hsp90 (**Figures 13C**).

In agreement with this experimental evidence, the evolutionary conserved CTD node Ile-538 in HtpG (corresponding to Ala-646 in Grp94 and Ala-577 in yeast Hsp90) emerged as a central element of the CTD regulatory motif involved in coordinating the inter-domain communications. The evidence of an efficient molecular communication between NTD and CTD regions at the interface is of special importance given a rapidly growing interest in developing novel and specific Hsp90 inhibitors targeting allosteric C-terminal nucleotide binding site [94]–[96]. Our findings may complement the recent study [96] by suggesting that binding of the C-terminal inhibitors to the Hsp90 region around the pivotal switch point may disrupt a dynamic equilibrium and block functional conformational changes required for normal progression of the ATPase cycle.

Hence, we identified universal functional motifs shared by the Hsp90 chaperones in different species that could play a universal role in coordinating functional dynamics and principal collective motions of Hsp90. These motifs may also serve as central functional regulators of Hsp90 activity, including control of ATP hydrolysis, the inter-domain communications and protein client binding. We observed that recognition sites may correspond to the conformationally mobile regions along slow modes of principal motions. Moreover, the recognition sites are often strategically positioned close to the regulatory anchor motifs containing catalytic and/or hinge residues. According to our results, functional coupling of structurally stable and conformationally mobile elements in the regulatory motifs is what may enable allosteric signaling and recognition of co-chaperones and protein clients. The central result of this study is that a small number of functional motifs may be utilized by the chaperone machinery to act collectively as central regulators of Hsp90 dynamics and activity, including the inter-domain communications, control of ATP hydrolysis, co-chaperone binding and protein client recognition. Mutations, drug targeting, and probing protein binding in these regions would allow furthering pinpointing specific residues responsible for various functions including protein binding.

## Discussion

It becomes more apparent now that functional dynamics of Hsp90 is closely associated with the ability of the molecular chaperone to bind and control activity of numerous protein clients. During client binding, the Hsp90 chaperone can adopt equilibrium between structurally different states that is maintained even after substrate loading [78], [79], [139]. To effectively sample Hsp90 functional states required for client loading, binding and release, nature has to provide a mechanism for efficient exploration of the conformational landscape. The results of our studies support the working hypothesis that molecular mechanisms of Hsp90 may be regulated by modulating conformational equilibrium of preexisting functional states via cooperative allosteric changes. According to this model, the molecular chaperone may employ a mechanism of conformational selection to control a dynamic equilibrium between different conformational states that can be switched towards functionally relevant complexes. Our findings corroborate with the recent results pointing to an important role of conformational selection mechanisms in a variety of biological systems [140]–[143].

In this work, we identified and characterized evolutionary and functionally conserved elements of the Hsp90 chaperone that may serve as key regulators of collective motions and hubs of long-range communication networks. We propose that functional clusters corresponding to stable regions in principal collective motions define minimally frustrated anchor sites (“hot spots”). These minimally frustrated residue clusters with optimized local interactions may be critical for regulation of allosteric signalling between distant protein regions. The proximal recognition sites (“soft spots”) may be functionally coupled to the anchoring regulatory residues and form networks of communicating interaction clusters. These “soft spots” may be formed by locally frustrated residues, whose interaction networks are energetically optimized and prone to dynamic modulation by mutations or protein client binding. Hence, allosteric signaling may involve a dynamic coupling between structurally rigid (minimally frustrated) and plastic (locally frustrated) clusters of residues. A switch in state at a soft cluster upon protein client binding can shift the thermodynamic balance and activate a specific network of allosterically coupled residues. This model is consistent with the central role of regulatory nodes as “discrete breathers” or independent dynamic segments in allosteric signaling [128], [144], [145]. The identified functional regulators of Hsp90 activity are typically situated at the inter-domain interfaces and are formed by co-evolving amino acid residues. As such, these motifs may also form so-called “protein sectors”, which are sparse residue clusters operating collectively and rather independently from each other [146], [147].

Overall, the proposed mechanistic picture of Hsp90 function is consistent with studies showing that allosteric networks orchestrating cooperative protein motions can be formed by evolutionary conserved and sparsely connected groups of residues, suggesting that rapid transmission of allosteric signals through a small network of distantly connected residue clusters may be a universal requirement encoded in the molecular chaperones and possibly other macromolecular assemblies. Our analysis has also confirmed that Hsp90 homologues sharing the same fold architecture may exhibit similar dynamical characteristics, including conservation of principal collective motions. The results support a mechanistic hypothesis according to which that the interaction networks regulating collective motions of the Hsp90 chaperone may be determined by the inherent structural architecture of the molecular chaperone. At the same time, the thermodynamics-based “conformational selection” of Hsp90 functional states is likely to be readily modulated by co-chaperones and protein binding partners.

Targeting allosteric binding sites and interrupting protein client binding with Hsp90 can produce a broad-spectrum antitumor activity and a low risk of drug resistance due to a combinatorial blockade of multiple signaling pathways. While there has been a steady progress in structural studies of the Hsp90-NTD complexes with small molecules, much less is known about the molecular basis of allosteric Hsp90 binding with co-chaperones and protein clients. A second nucleotide-binding domain has been reported to exist in the C-terminus of Hsp90, and specific inhibitors to this domain are represented by novobiocin and the related coumarin antibiotics [80]–[82]. Interrupting protein client binding with Hsp90 provides a different therapeutic approach, in which the Hsp90 activity may be unaffected and specific client proteins prevented from binding to the Hsp90 complex. Chemical genomics and gene expression-based approaches have identified novel modulators of Hsp90 activity, including celastrol [148]. This Hsp90 modulator does not interfere with ATP binding but may allosterically intervene in Hsp90 binding with co-chaperones [149]. It was proposed that celastrol may interact with the C-terminal portion of Hsp90 [150]. According to the alternative view, celastrol may not bind to Hsp90 but could rather interact with the N-terminal of protein kinase clients and the middle Hsp90-binding domain of Cdc37 [151].

The results of our study could guide design of experiments for probing allosteric binding of celastrol by introducing mutations in the communicating residues from the regulatory and recognition motifs of Hsp90. Integration of computational and experimental approaches may thus facilitate experimentally-guided mapping of allosteric binding states and help to specify whether Hsp90 or its co-chaperone Cdc37 is the primary target for binding of celastrol. Recent evidence suggests that Hsp90 interacts and stabilizes a growing list of protein kinases, and Cdc37 serves as the key co-chaperone for a large portion of the yeast kinome [152]. Hsp90 and Cdc37 are both required for activity and stability of many tumor-inducing signaling protein kinases, and tumors appear to become addicted to these chaperones. Deregulation of multiple signaling kinases in cancer kinome may be effectively suppressed by intervening with the chaperoning function of Hsp90-Cdc37. Novel inhibitors that disrupt Cdc37-Hsp90 interactions may thus hold promise as potential therapeutics against a variety of kinase-dependent cancers. Continuous integration of computational and experimental investigations of Hsp90 into a system biology-based platform may be useful for probing mechanisms of molecular chaperones and guiding discovery of allosteric Hsp90 modulators.

## Materials and Methods

### MD Simulations

MD simulations of the Hsp90 structures (each of 20 ns duration) were performed for a closed ATP-bound conformation of yeast Hsp90 (crystal structure) (PDB ID 2CG9) [54]; a “V-shaped” conformation of the mammalian Grp94 homologue from complexes with ADP (PDB ID 2O1V) and AMP-PNP (PDB ID 2O1U) (crystal structure) [56]; an open Apo form of the bacterial homologue HtpG (PDB ID 2IOQ) (crystal structure) [55]; a semi-closed, ADP-bound form of the bacterial homologue HtpG (PDB ID 2IOP) (crystal structure) [55]; and an extended Apo HtpG conformation (solution structure from SAXS studies) [61]. All crystallographic water molecules, bound inhibitors, and other heteroatoms were removed. The retrieved structures were examined for missing and disordered residues. The missing residues and unresolved structural segments were modeled using the program MODELLER which is an automated approach to comparative protein structure modeling by satisfaction of spatial restraints [153], [154]. MD simulations were carried out using NAMD 2.6 [155] with the CHARMM27 force field [156], [157] and the explicit TIP3P water model as implemented in NAMD 2.6 [158]. The VMD program was used for the preparation and analysis of simulations [159], [160]. The employed MD protocol was described in full details in our earlier studies [161]–[163]. In brief, structures were solvated in a water box with the buffering distance of 10 Å. Assuming normal charge states of ionizable groups corresponding to pH 7, sodium (Na^+^) and chloride (Cl^−^) counter-ions were added to achieve charge neutrality in MD simulations at physiological concentration of 0.15 mol/L. All Na^+^ and Cl^−^ ions were placed at least 8 Å away from any protein atoms and from each other. The system was subjected to initial minimization for 20,000 steps (40 ps) keeping protein backbone fixed which was followed by 20,000 steps (40 ps) of minimization without any constraints. Equilibration was done in steps by gradually increasing the system temperature in steps of 20 K starting from 10 K until 310 K and at each step 15000 steps (30 ps) equilibration was run keeping a restraint of 10 Kcal mol−1 Å−2 on protein alpha carbons (C_α_). Thereafter the system was equilibrated for 150,000 steps (300 ps) at 310 K (NVT) and then for further 150,000 steps (300 ps) at 310 K using Langevin piston (NPT) to achieve uniform pressure. Finally the restrains were removed and the system was equilibrated for 500,000 steps (1 ns) to prepare the system for simulation. An NPT simulation was run on the equilibrated structure for 20 ns keeping the temp at 310 K and pressure at 1 bar using Langevin piston coupling algorithm. The integration time step of the simulations was set to 2.0 fs, the SHAKE algorithm was used to constrain the lengths of all chemical bonds involving hydrogen atoms at their equilibrium values and the water geometry was restrained rigid by using the SETTLE algorithm. Nonbonded van der Waals interactions were treated by using a switching function at 10 Å and reaching zero at a distance of 12 Å. The particle-mesh Ewald algorithm (PME) as implied in NAMD was used to handle long range electrostatic forces.

### Principal Component Analysis

Protein flexibility was analyzed by combining the results of MD simulations with the principal component analysis of conformational ensembles [164], [165]. The covariance matrix between residues (represented by the C_α_ atoms) i and j was calculated for each of the 20 ns MD simulation trajectories: snapshots from trajectories were taken every 200 ps, overall translation and rotation were removed, and only C_α_ was kept for analysis.

To obtain collective motion coordinates that represent the overall dynamics of each trajectory, PCA was performed, in which the covariance matrix 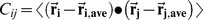 was diagonalized to yield a set of eigenvectors and eigenvalues. The correlation matrix 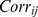 represents the correlation between the motion of atom i and of atom j, obtained from the normalization of the covariance matrix.
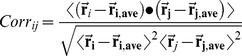
The eigenvectors represent the directions in the multidimensional space that correspond to independent modes of atomic motion, while the eigen values represent their corresponding amplitudes [164], [165]. The principal components of protein motions are analyzed by projecting MD trajectories onto directions corresponding to the largest eigen vectors. The correlation value is the normalized covariance matrix, ranging from −1 to 1. The calculations were performed using the CARMA package [166] and PCA_NEST web-based service [167].

## Supporting Information

Figure S1 **Time-Dependent History of High Occupancy Salt Bridges.** Thermal fluctuations of the salt bridges in Grp94 (**A**) and yeast Hsp90 (**B**). (**A**) The depicted high occupancy hydrogen bond interactions in the M-domain and CTD of Grp94 are Glu564-Lys530 (in blue), Asp573-Lys493 (in red), and Glu548-Arg660 (in green). (**B**) The depicted high occupancy hydrogen bond interactions in yeast Hsp90 are Glu165-Arg174 (NTD, in blue), Glu355-Lys335 (CTD, in red), and Asp506-Lys426 (M-domain/CTD interface, in green).
(TIF)

Figure S2 **Allosteric Coupling of the Regulatory and Recognition Sites in the Hsp90 Chaperone.** The coordinated involvement of the inter-domain charged linker, the catalytic functional loop from the M-domain and the projecting recognition loops are shown in blue spheres (only main chain is shown). The ADP-bound Hsp90 crystal structure (PDB ID 2IOP) was used for clarity of illustration. The protein structure is shown in ribbons colored according to secondary structures.
(TIF)
