# Supplementary figures and images for: Probing Molecular Mechanisms of the Hsp90 Chaperone: Biophysical Modeling Identifies Key Regulators of Functional Dynamics
Source: PLoS One. 2012 May 18;7(5):e37605. doi: 10.1371/journal.pone.0037605 (PMC3356286; doi:10.1371/journal.pone.0037605)

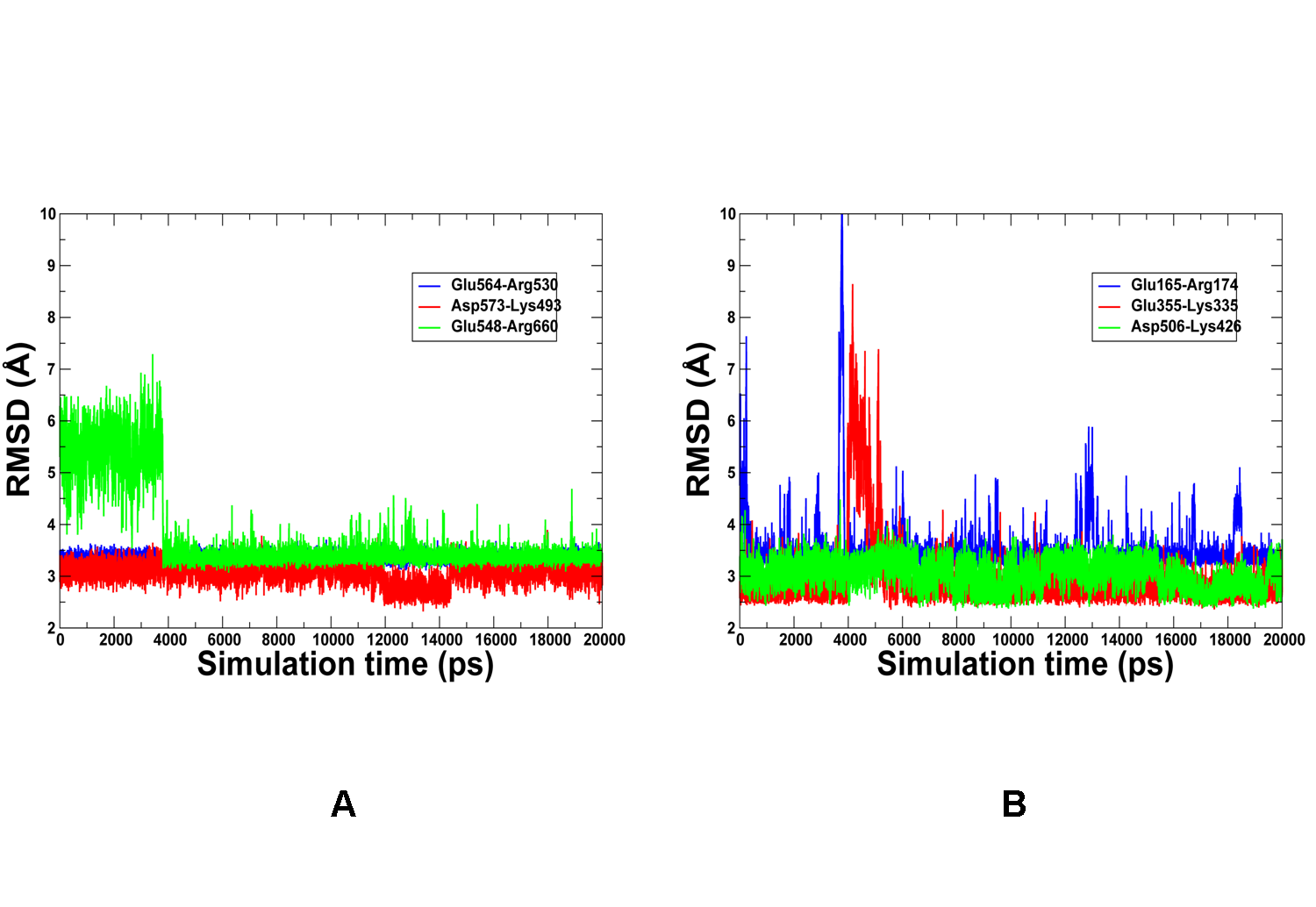

Supplement: Figure S1 — Time-Dependent History of High Occupancy Salt Bridges. Thermal fluctuations of the salt bridges in Grp94 (A) and yeast Hsp90 (B). (A) The depicted high occupancy hydrogen bond interactions in the M-domain and CTD of Grp94 are Glu564-Lys530 (in blue), Asp573-Lys493 (in red), and Glu548-Arg660 (in green). (B) The depicted high occupancy hydrogen bond interactions in yeast Hsp90 are Glu165-Arg174 (NTD, in blue), Glu355-Lys335 (CTD, in red), and Asp506-Lys426 (M-domain/CTD interface, in green). (TIF) [file pone.0037605.s001.tif]

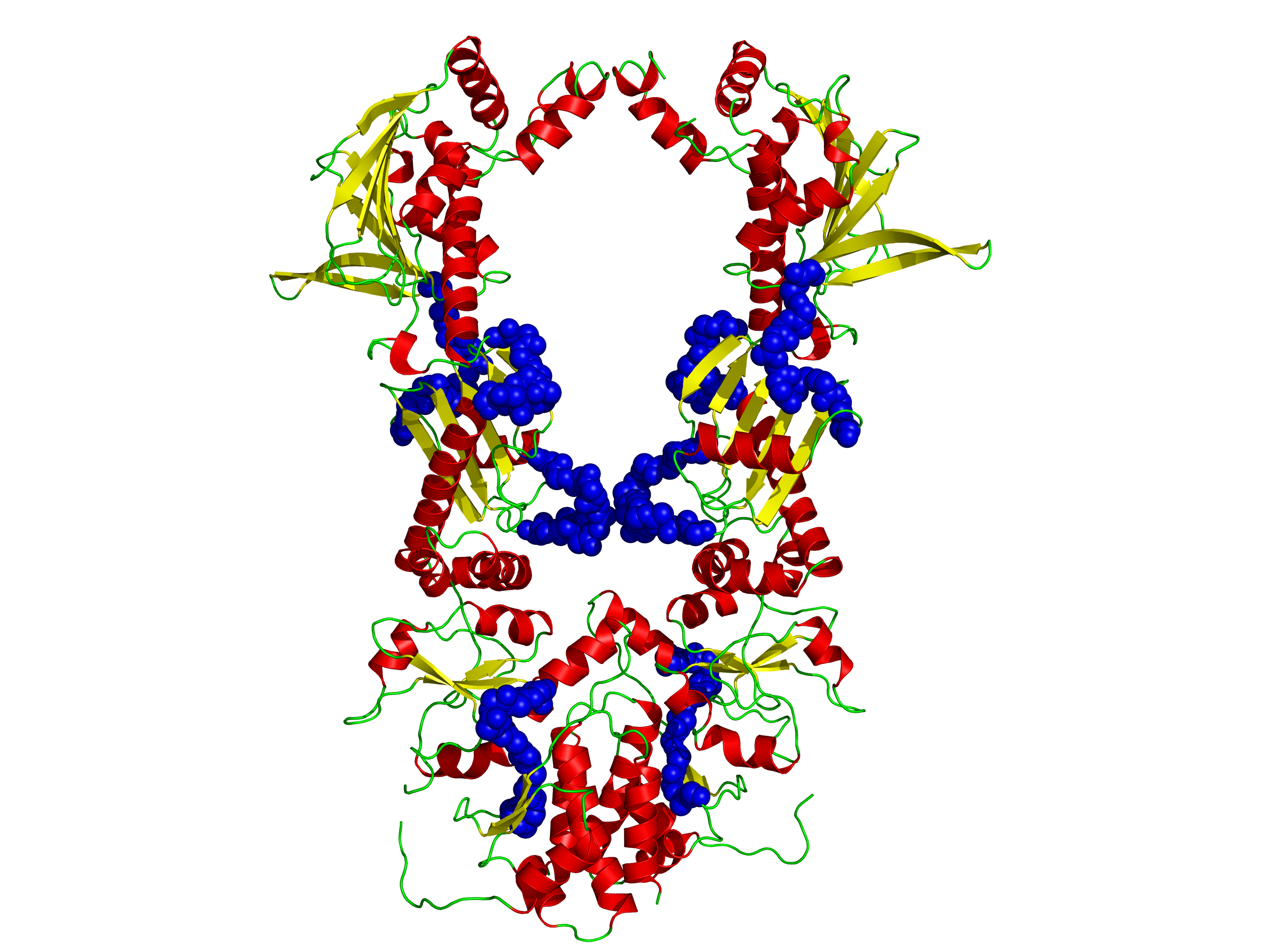

Supplement: Figure S2 — Allosteric Coupling of the Regulatory and Recognition Sites in the Hsp90 Chaperone. The coordinated involvement of the inter-domain charged linker, the catalytic functional loop from the M-domain and the projecting recognition loops are shown in blue spheres (only main chain is shown). The ADP-bound Hsp90 crystal structure (PDB ID 2IOP) was used for clarity of illustration. The protein structure is shown in ribbons colored according to secondary structures. (TIF) [file pone.0037605.s002.tif]
